# Investigation of community pharmacists’ knowledge and attitudes of pharmacogenomics testing: implication for improved pharmacogenomic testing practice

**DOI:** 10.1186/s40246-024-00574-z

**Published:** 2024-01-30

**Authors:** Azza Ramadan, Anan S. Jarab, Ahmad Z. Al Meslamani

**Affiliations:** 1 Department of Pharmaceutical Sciences, College of Pharmacy, Al Ain University, 112612 Abu Dhabi, United Arab Emirates; 2 AAU Health and Biomedical Research Center, Al Ain University, Abu Dhabi, United Arab Emirates; 3 Department of Clinical Pharmacy, Faculty of Pharmacy, Jordan University of Science and Technology, P.O. Box 3030, Irbid, 22110 Jordan

**Keywords:** Pharmacist, Pharmacogenomics testing, Pharmacogenetics, UAE

## Abstract

**Background:**

Community pharmacists must be well-equipped to advance pharmacogenomics services. Nevertheless, limited data is available regarding pharmacists' knowledge and attitudes toward pharmacogenomics testing. The present study aimed to evaluate community pharmacists' knowledge and attitudes toward pharmacogenomics testing in the UAE.

**Methods:**

In this cross-sectional study, a validated, online, self-administered survey, was randomly distributed to community pharmacists across the United Arab Emirates (UAE).

**Results:**

The participants demonstrated poor knowledge about pharmacogenomic testing (median score < 8). Having 10–29 (Adjusted odds ration [AOR]: 0.038; 95% CI: 0.01–0.146, *p* = 0.001) and 30–49 (AOR: 0.097; 95% CI: 0.04–0.237, *p* = 0.001) patients per day was associated with poorer knowledge. Also, receiving 10–29 (AOR: 0.046; 95% CI: 0.005–0.401, *p* = 0.005), 30–49 (AOR: 0.025; 95% CI: 0.003–0.211, *p* = 0.001), and > 50 (AOR: 0.049; 95% CI: 0.005–0.458, *p* = 0.008) prescriptions decreased the odds of having good knowledge. Around half (43.9%) of the participants did not show a positive attitude toward pharmacogenomic testing (median score < 11). Having 30–49 patients per day (AOR: 5.351; 95% CI: 2.414–11.860, *p* = 0.001) increased the odds of good knowledge while receiving 10–29 (AOR: 0.133; 95% CI: 0.056–0.315, *p* = 0.001) and 30–49 (AOR: 0.111; 95% CI: 0.049–0.252, *p* = 0.001) prescriptions a day were associated with decreased odds of positive attitude toward the pharmacogenomics testing.

**Conclusions:**

The findings indicate a lack of knowledge and less-than-ideal attitudes among community pharmacists regarding pharmacogenomics testing. Enhanced efforts focused on educational initiatives and training activities related to pharmacogenomics testing is needed. Additionally, reducing workload can facilitate better knowledge acquisition and help mitigate unfavorable attitudes.

## Introduction

In recent years, precision or personalized medicine has become increasingly important in healthcare and research to improve health outcomes [1]. Since genetic variability appears to determine drug responses, the concept of pharmacogenomics testing is gaining importance. Pharmacogenomics testing combines the principles of pharmacology and genomics to improve an individual's response to a drug by tailoring the medication and doses according to hereditary characteristics related to drug distribution, metabolism, and excretion [2].

It is estimated that as much as 90–95% of a drug response variability may be attributed to pharmacogenomics [3, 4]. Indeed, moderate response to therapy has been attributed to allele variations and their differences across populations, which also influence adverse drug reactions. Hence, pharmacogenomics testing can obliterate inappropriate drug use and improve safety profile by adjusting therapy based on inter individual genetic variations and is a topic of great interest in diverse medical professions (physicians, pharmacists, nurses). Clinical trials attest that incorporating pharmacogenomic information to guide treatments improves clinical outcomes, as in the case of major depressive disorder [5].

Community pharmacists are among the most accessible healthcare professionals and constitute one of the largest professional groups worldwide, alongside physicians and nurses. They serve as essential agents in disseminating pharmacogenomics services within their respective communities, playing a vital role in delivering these services.

Indeed, to date, pharmacogenomics testing services in community pharmacies have been incorporated and evaluated in the USA, Canada, and the Netherlands [6]. Nevertheless, the knowledge and practice of pharmacogenomics among pharmacists are generally low, and difficulties exist in interpreting results, as reported in a systematic review and pilot study by Hansen et al. [7], and Yau et al. [8], respectively.

Of note, in the United Arab Emirates (UAE), a high proportion of genetic disorders and genetic variants have been reported [9, 10]. Thus, pharmacogenomics is essential in designing therapeutic measures and calls for relevant policies for its implementation. A previous study in the UAE among a diverse group of healthcare workers comprising pharmacists, nurses, physicians, managers, and allied health professionals reported a fair pharmacogenomics knowledge higher in males than females [11]. Some reported barriers to implementation were cost and lack of training and education. Currently, there is limited research within the field of pharmacogenomics testing among community pharmacists in the UAE.

Therefore, this study aimed to assess community pharmacists' knowledge and attitudes toward utilizing pharmacogenomics testing in the UAE. The present study's findings provide insight into knowledge gaps and attitudes to identify the barriers and enablers to implementing community pharmacy-based pharmacogenomics testing services.

## Methods

### Study design and subjects

In the current cross-sectional study, an online self-administered survey was randomly distributed via convenience sampling technique in English using Google forms to community pharmacists across the UAE from May to July 2023. The survey included a statement informing the participants that completing the survey represents a consent to participate. The study received ethical approval from Al Ain University (Reference: COP/AREC/AD/41).

### Sample size calculation

The sample size was calculated using a Raosoft sample size calculator, a confidence limit of 5%, and a 95% confidence interval estimate of the proportion. A minimum sample size of 384 was needed. The study included both locals (Emiratis) and expats (residents).

### Study instrument

After extensive literature review, the present study survey was adopted from earlier research implemented for similar purposes [12] and changes were made where appropriate. These changes included refining the questionnaire by providing insights on the aspects of pharmacogenomics in pharmacy practice, developing items that were not originally included in the instrument, rewording and sometimes excluding already existing items and altering the flow of the items in both knowledge and attitude parts. Next, an expert panel including two academic professors in pharmacogenomics and a genetics scientific researcher evaluated the questionnaire for face and content validity and ensured that it was not solely based on theoretical knowledge but also reflected real-world applications of pharmacogenomics and in alignment with the study objectives. Next, the questionnaire was piloted on ten pharmacists to ensure its clarity and comprehensiveness. Findings from the pilot study were not included in the final analysis. The survey started with a brief introduction that described the study objectives, emphasized the confidentiality of the participants, and informed them that completing the survey represents consent to participate in the study. The survey included socio-demographic questions, questions on education and practice, ten knowledge questions using a yes/no scale, and fourteen questions assessing attitudes toward pharmacogenomics testing on a 5-point Likert scale ranging from strongly disagree (1 point) to strongly agree (5 points).

### Data analysis

Data analysis was conducted using the Statistical Package for the Social Sciences (SPSS), version 26. Categorical results were expressed as absolute numbers accompanied by percentages, while continuous variables were presented as medians with their interquartile ranges (IQR). To compute the scores for knowledge and attitude, responses were initially coded as 1 for correct or favorable answers and 0 for incorrect or unfavorable answers. Total knowledge scores were calculated based on the number of correct responses. Using the median score as a reference point for both the knowledge and attitude, scores were classified into two categories: “good knowledge” versus “poor knowledge” and “positive attitude” versus “negative attitude”. Initially, univariate analysis was performed to examine the relationship between each independent variable of knowledge and attitude and the dependent variables of knowledge and attitudes. Variables with a *P* value less than 0.10 were then included in the logistic regression analysis to construct models identifying variables significantly and independently associated with knowledge and attitudes. A *p*-value of less than 0.05 was deemed statistically significant to confirm which variables are substantially associated with the outcomes.

## Results

There were 400 community pharmacists who completed the survey, of which 399 were included in the data analysis, and one participant was excluded because of missing responses. Results showed that 222 (55.6%) were females, 215 (53.9%) were aged between 27 and 35 years, and 54 (13.5%) had more than ten years of experience (Table 1). Results showed that 151 (37.8%) participants reported counseling a customer about pharmacogenomics testing.

**Table 1:** Characteristics of the study participants (*N* = 399)

Parameters	Total, *n* (%)
*Gender*	
Female	222 (55.6%)
Male	177 (44.4%)
*Age (years)*	
22–26	84 (21.1%)
27–35	215 (53.9%)
36–45	85 (21.3%)
> 45	15 (3.8%)
*Years of experience*	
< 2	49 (12.3%)
2–5	155 (38.8%)
6–10	141 (35.3%)
> 10	54 (13.5%)
*Educational level*	
BPharm (Bachelor in pharmacy)	371 (93.0%)
PharmD (Doctor in pharmacy)	7 (1.8%)
Graduate (Master or PhD)	12 (3.0%)
Diploma	9 (2.3%)
*Type of pharmacy working in*	
Chain	278 (69.7%)
Independent	121 (29.3%)
*Average number of patients per day*	
< 10	19 (4.8%)
10–29	54 (13.5%)
30–49	149 (37.3%)
≥ 50	177 (44.4%)
*Average prescriptions per day*	
< 10	122 (30.6%)
10–29	146 (36.6%)
30–49	44 (11.0%)
≥ 50	87 (21.8%)
*Work hours per week*	
40–50	347 (87.0%)
< 40	21 (5.3%)
> 50	31 (7.8%)
*Average time spent with each patient (minute)*	
< 2	3 (0.8%)
2–5	67 (16.8%)
> 5	329 (82.5%)
*Workload in the pharmacy*	
Very low	10 (2.5%)
Low	20 (5.0%)
Moderate	267 (66.9%)
High	74 (18.5%)
Very high	28 (7.0%)
*Counseled a customer about pharmacogenetics test results before*	
Yes	151 (37.8%)
No	248 (62.2%)
*Received a formal education about pharmacogenetics*	
Yes	258 (64.7%)
No	141 (35.3%)
*Attended training workshops on pharmacogenetics*	
Yes	144 (36.1%)
No	255 (63.9%)

### Knowledge of pharmacogenomics

**Fig. 1:**
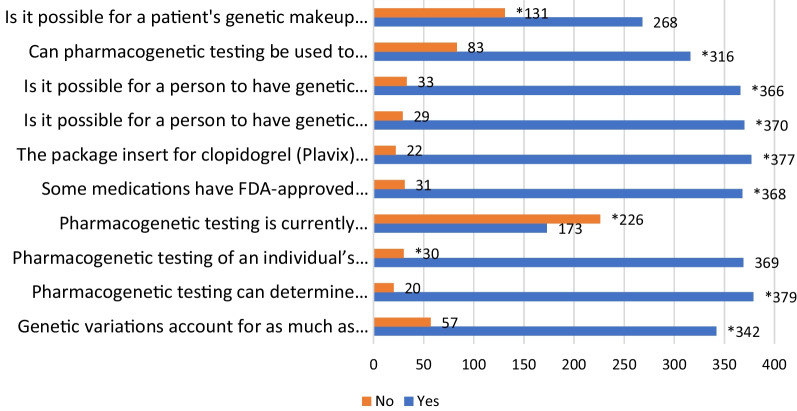
Knowledge of Participants about pharmacogenomics testing (*indicates correct answers)

Of the 399 participants included in the study, 291 (73%) had poor knowledge (median score < 8) about pharmacogenomics testing. As shown in Fig. 1, the most common knowledge gaps were that 226 (56.6%) participants were not aware that pharmacogenomics testing is not available for all medications. Almost two-third, 131 (67.1%), did not know that a patient's genetic makeup cannot change over time, and hence the patient’s response to medications will not change over their lifetime, and 83 (20.8%) participants did not know that identification of potential drug–drug interaction based on a patient's profile is possible through pharmacogenomics testing.

Having 10–29 (AOR: 0.038; 95%CI: 0.01–0.146, *p* = 0.001) and 30–49 (AOR: 0.097; 95%CI: 0.04–0.237, *p* = 0.001) patients per day decreased the likelihood of having good knowledge. Moreover, receiving 10–29 (AOR: 0.046; 95%CI: 0.005–0.401, *p* = 0.005), 30–49 (AOR: 0.025; 95%CI: 0.003–0.211, *p* = 0.001), and > 50 (AOR: 0.049; 95%CI: 0.005–0.458, *p* = 0.008) prescriptions decreased the odds of having good knowledge (Table 2).

**Table 2:** Association of pharmacist characteristics with knowledge (good vs. poor) (*N* = 399)

Independent variables (variables vs reference)	Knowledge (Good vs poor)	
	AOR^a^ (95% CI^b^)	*P* value
*Years of experience*		
2–5 vs < 2	4.369 (0.850–22.443)	0.077
6–10 vs < 2	1.809 (0.506–6.468)	0.362
> 10 vs < 2	1.802 (0.559–5.811)	0.324
*Pharmacy type*		
Independent vs chain	0.616 (0.346–1.095)	0.099
*Average number of patients per day*		
10–29 vs < 10	0.038 (0.01–0.146)	0.001***
30–49 vs < 10	0.097 (0.04 -0.237)	0.001***
≥ 50 vs < 10	0.636 (0.320–1.224)	0.196
*Average prescriptions per day*		
10–29 vs < 10	0.046 (0.005–0.401)	0.005**
30–49 vs < 10	0.025 (0.003–0.211)	0.001***
≥ 50 vs < 10	0.049 (0.005–0.458)	0.008**
*Workload in the pharmacy*		
High vs average	0.712 (0.123–4.136)	0.705
Very high vs average	0.571 (0.091–3.591)	0.550
Low vs average	0.983 (0.120–8.031)	0.987
Very low vs average	0.471 (0.044–5.004)	0.533
*Received a formal education about pharmacogenetics*		
Yes vs no	0.530 (0.261–1.076)	0.079

^a^AOR: adjusted odds ratio

^b^CI: confidence interval

^**^P-value ≤ 0.01, ***P-value ≤ 0.001

### Attitude toward pharmacogenomic

Among the study participants, 175 (43.9%) did not have a positive attitude toward pharmacogenomic testing (median score < 11). Only a small proportion of the study sample, 26 (6.6%), disagreed that pharmacogenomics testing is more important in the hospital rather than the community pharmacy setting (Table 3). Additionally, only 31 (7.8%) participants disagreed that interpreting the pharmacogenomics testing information was difficult. Furthermore, 278 (69.7%) of the pharmacists agreed that pharmacogenomics testing is not compatible with their personal values. Additionally, the majority of pharmacists were concerned about unauthorized access to test results (74.7%) and the effects of the test results on eligibility for private insurance (72.9%) **(**Table 3**)**.

**Table 3:** Attitude toward pharmacogenetic testing

Item	Strongly agree	Agree	Neither	Disagree	Strongly disagree
Pharmacogenetic testing is a promising innovation in medicine and drug therapy	223 (55.9%)	137 (34.3%)	17 (4.3%)	17 (4.3%)	5 (1.3%)
Pharmacogenetic testing can offer a useful tool to the way I usually recommend medications	201 (50.4%)	159 (39.8%)	18 (4.5%)	14 (3.5%)	7 (1.8%)
I will utilize pharmacogenetic test results when practice guidelines for the use and interpretation of these tests are available	198 (49.6%)	158 (39.6%)	19 (4.8%)	20 (5.0%)	4 (1.0%)
Pharmacogenetic testing is advantageous in case of non-response to an essential drug (e.g.,, analgesic)	232 (58.1%)	129 (32.3%)	14 (3.5%)	19 (4.8%)	5 (1.3%)
Pharmacogenetic testing is more important in the hospital setting rather than the community pharmacy setting	189 (47.4%)	167 (41.9%)	17 (4.3%)	19 (4.8%)	7 (1.8%)
The interpretation of the pharmacogenetic testing information is difficult	166 (41.6%)	175 (43.9%)	27 (6.8%)	26 (6.5%)	5 (1.3%)
Pharmacogenetic testing will potentially help decrease the number of adverse drug events	168 (42.1%)	146 (36.6%)	43 (10.8%)	37 (9.3%)	5 (1.3%)
Pharmacogenetic testing will potentially help decrease the risk of drug–drug interaction	136 (34.1%)	134 (33.6%)	59 (14.8%)	63 (15.8%)	7 (1.8%)
Pharmacogenetic test results should be utilized when information about the test is included in the package inserts	197 (49.4%)	161 (40.4%)	16 (4.0%)	19 (4.8%)	6 (1.5%)
Patients should be educated about the purpose, benefits, limitations, and risks of pharmacogenetic testing	175 (43.9%)	166 (41.6%)	27 (6.8%)	25 (6.3%)	6 (1.5%)
I am concerned that unauthorized personnel may gain access to the results of the pharmacogenetic test	144 (36.1%)	154 (38.6%)	31 (7.8%)	61 (15.3%)	9 (2.3%)
I am concerned about the effect of the test results on my patients' eligibility for private health insurance	152 (38.1%)	139 (34.8%)	35 (8.8%)	59 (14.8%)	14 (3.5%)
I will be reluctant to adopt/utilize pharmacogenetic test results until I see it working for patients	223 (55.9%)	139 (34.8%)	13 (3.3%)	19 (4.8%)	5 (1.3%)
Pharmacogenetic testing is not compatible with my personal values	140 (35.1%)	138 (34.6%)	28 (7.0%)	81 (20.3%)	12 (3.0%)

The logistic regression analysis showed that having 30–49 patients per day (AOR: 5.351; 95%CI: 2.414–11.860, *p* = 0.001) increased the odds of having good knowledge. On the other hand, receiving 10–29 (AOR: 0.133; 95%CI: 0.056–0.315, *p* = 0.001) and 30–49 (AOR: 0.111; 95%CI: 0.049–0.252, *p* = 0.001) prescriptions a day were associated with decreased odds of having a positive attitude toward the pharmacogenomics testing. (Table 4**)**.

**Table 4:** Association of pharmacist characteristics attitude (positive vs. negative) (*N* = 399)

Independent variables (variables vs reference)	Attitude (Positive vs poor)	
	AOR^a^ (95% CI)^b^	*P* value
*Gender*		
Male vs Female	1.359 (0.855–2.158)	0.194
Pharmacy type		
Independent vs chain	1.517 (0.920–2.501)	0.102
*Average number of patients per day*		
10–29 vs < 10	3.008 (0.961–9.415)	0.167
30–49 vs < 10	5.351 (2.414–11.860)	0.001***
≥ 50 vs < 10	1.102 (0.630–1.930)	0.733
*Average prescriptions per day*		
10–29 vs < 10	0.133 (0.056–0.315)	0.001***
30–49 vs < 10	0.111 (0.049–0.252)	0.001***
≥ 50 vs < 10	0.408 (0.149–1.118)	0.081
*Work hours per week*		
Less than 40 vs 40–50	1.968 (0.836–4.634)	0.121
More than 40 vs 40–5	0.381 (0.099–1.464)	0.160
*Received a formal education about pharmacogenetics*		
Yes vs no	1.228 (0.679–2.221)	0.496
*Attended training workshops on pharmacogenetics*		
Yes vs no	1.021 (0.616–1.691)	0.936

^a^AOR: adjusted odds ratio

^b^CI: confidence interval

^***^P-value ≤ 0.001

## Discussion

In this study, less than one-third of the community pharmacists had sufficient knowledge of pharmacogenomics testing, while almost half did not have a positive attitude toward the test. Poor knowledge was associated with the increased number of patients and prescriptions daily. A positive attitude toward pharmacogenomics was associated with an increased number of patients, while a negative attitude was associated with an increased number of daily prescriptions.

This study underscores a significant knowledge and attitude gap among community pharmacists regarding pharmacogenomics testing, a cornerstone of personalized medicine. Such gaps in knowledge and suboptimal attitudes can hinder the successful implementation of pharmacogenomics testing in community pharmacies. When equipped with enhanced knowledge and a positive attitude toward pharmacogenomics testing, Community Pharmacists can play a pivotal role in optimizing drug therapy and enhancing patient outcomes. Given the observed correlation between limited knowledge and an increase in the number of patients and prescriptions per day, it is imperative to employ effective time management and workload balancing measures. This will ensure that pharmacists have the opportunity for continuous learning and professional development.

This study's finding of inadequate overall knowledge about pharmacogenomics testing among most pharmacists in this study corroborates earlier research findings. A qualitative study reported poor self-rated knowledge of genomics and pharmacogenomics among pharmacists working in tertiary hospitals, health clinics and community pharmacies in the UAE, where most of the participants indicated being unaware of testing services in the UAE [13]. Similarly, over two-thirds of the community pharmacists had poor clinical knowledge of pharmacogenomics and genetics, and the knowledge score differed according to the educational degree in Saudi Arabia. Also, in a study of US pharmacists, the overall subjective self-reported knowledge, i.e.,, the understanding of pharmacogenomics, was rated as fair or poor (83%) [15].

Surprisingly, the knowledge score of pharmacogenomic-related questions was poor among hospital pharmacists in Jeddah, Saudi Arabia [16]. Furthermore, in another Saudi Arabia study, 69% of healthcare professionals specializing in medicine, nursing, and pharmacy had poor knowledge about personalized medicine [17]. Around 60% of Syrian respondents, the majority of which were pharmacists and physicians, were unaware of the Clinical Pharmacogenetics Implementation Consortium (CPIC) nor the implication of Poor Metabolizer Phenotype on drug metabolism [18]. On the other hand, the overall knowledge of pharmacogenomics was rated moderate among the community pharmacists in Nigeria, which could be explained by the participants having previous pharmacogenomics training and postgraduate qualifications such as PharmD [19]. Interestingly, over two-thirds were unaware that clopidogrel and pantoprazole need pharmacogenomics testing. Another study reported good knowledge about pharmacogenomics testing in Indonesia among community pharmacists [20]. Overall, the diversity in knowledge could be attributed to the difference in the investigated population and the instruments used to evaluate knowledge.

The most common areas of poor knowledge were the lack of knowledge that pharmacogenomics testing is currently unavailable for all medications, the possibility for a patient's genetic makeup not changing over time and the possibility of identifying the potential drug–drug interactions through pharmacogenomics testing.

The deficiency in knowledge regarding pharmacogenomics is predominantly attributed to insufficient education on the subject; examples of such ignorance include the unavailability of genetic tests for all medications and an individual's genetic makeup remains unaltered over time. Pharmacists might be uninformed about the limitations of pharmacogenomics testing, rendering them oblivious to the fact that not all medications, including those available over the counter, have corresponding genetic tests. Rapid advancements in pharmacogenomics may surpass the updates made to educational curricula, resulting in knowledge gaps about novel developments, such as genetic testing for drug–drug interactions. Limited exposure to the practical applications of pharmacogenomics testing in professional settings exacerbates these knowledge gaps. For instance, a pharmacist in a community setting might not encounter pharmacogenomics testing as frequently as one stationed in a specialized research hospital. This leads to a lack of understanding regarding its applications in assessing drug interactions and patient genetic variability. These findings underscore the importance of adequate training and integration of more comprehensive pharmacogenomics courses in the pharmacy curriculum, which was proved to be associated with poor knowledge about pharmacogenomics testing in earlier studies [21, 22].

There is a need for concerted efforts toward educational and training activities, particularly regarding pharmacogenomics testing for medications available in the UAE, the influence of the non-changing genetic makeup on response to medications, and the indispensable use of pharmacogenomics in identifying potential drug–drug interactions. Indeed, a study has shown that pharmacogenomics knowledge was significantly greater among pharmacists who were educated and trained about pharmacogenomics and counseled patients regarding their pharmacogenomics results [23]. Also, to assess the impact of continuing education programs, Hayashi et al. [24] evaluated the effect of didactic, i.e., traditional teacher-based learning, and case-based studies-related programs via synchronous (virtual) and asynchronous (online self-study) environments on Canadian pharmacists' confidence in pharmacogenomics and their level of knowledge. It is noteworthy to mention that 88.9% of the participants were community pharmacists. The study found that the pharmacist's confidence in pharmacogenomics improved, evidenced by the median change of the "disagree" responses pre-program to "agree" post-program. Remarkably, their pharmacogenomics mean knowledge was increased from 20.8% pre-program to 70.2% post-program. Country-wide educational programs and policies are crucial interventions demonstrated to be effective in previous studies [25, 26]. For instance, using a testing 'toolkit' among US community pharmacists facilitates the delivery of pharmacogenomics testing services by incorporating effective pharmacist-patient communication and strategies to improve understanding of tests. More studies are needed to determine the effectiveness of these interventions.

In this study, knowledge was inversely associated with both the number of individuals seen per day and the number of prescriptions per day. This could be explained by the high workload manifested by the increased number of patients and prescriptions and, hence, less time for professional development activities (less exposure) such as workshops and seminars.

It was observed that the main hindrance to engaging in continuing education activities/programs among Saudia Arabia pharmacists was the lack of personal time. Furthermore, these pharmacists favored seminars rather than conferences and reading journal articles as a learning tool [27]. Also, Indonesian hospital and community pharmacists who attended continuing professional development programs had better pharmacogenomics knowledge than non-attendants [28].

Workload impact needs to be considered as it could reduce healthcare professionals' ability and the hours devoted to continuing education for pharmacists, adversely affecting their knowledge. In an Indonesian study, reduced pharmacist workload was associated with reallocating pharmacy time to provide prescription suggestions and decrease dispensing errors [29]. By extension, a reduced workload can facilitate the time needed for knowledge acquisition about pharmacogenomics testing. Continuous efforts to maintain an appropriate workload for pharmacists are recommended to ensure ongoing professional development.

Results showed that about half of the participants held negative attitudes toward pharmacogenomic testing. Consistent with earlier research findings [13, 14, 30], the present study participants demonstrated mixed attitudes toward pharmacogenomic testing. In contrast, more than two-thirds of the participants in Kuwait had positive attitudes toward pharmacogenomics testing and its clinical implications, more so among pharmacists than physicians [22]. Similarly, positive attitudes were manifested by willingness for pharmacogenomics testing services, interpretation of pharmacogenomics test results, and making subsequent recommendations by the pharmacists in Nigeria [19]. In a Syrian study, most of the participants, including pharmacists and physicians, were willing to provide pharmacogenomics counseling before medication dispensing and in support of conducting the test to predict drug efficacy before medication prescription [18]. These study findings, which suggest positive attitudes, contradict the present study results, where most of the pharmacists indicated that interpreting the pharmacogenomics testing information is difficult and incompatible with their personal values. In addition, the pharmacists in this study did not believe that pharmacogenomics testing in the community pharmacy setting is as essential as in the hospital setting.

Though there are fewer studies on pharmacists, a study on US physicians reported improved attitudes toward pharmacogenomics testing and clinical decision-making following a brief hour-long pharmacogenomics education program [31]. This evidence suggests that educational programs targeting community pharmacists could be implemented and evaluated. More importantly, it is imperative that these programs are tailored to the community rather than hospital pharmacists. For instance, these programs can include demonstrating how the test will be conducted and recommending medications to the prescriber to amend their prescription based on the genetic testing results. Indeed, recent studies are evaluating the delivery of pharmacogenomic testing in a community pharmacy setting [6, 32, 33]. Engaging and involving these pharmacists in these educational programs will emphasize their vital contribution to medication decision-making and improving patient's health-related outcomes. Our study highlighted the concerns among community pharmacists regarding unauthorized access to patients' results and the patient's eligibility for insurance. Similarly, Indonesian hospital pharmacists were concerned about pharmacogenomics test reimbursement, i.e., financial coverage, privacy issues relating to unauthorized access, and discrimination in employment or healthcare system based on the test results. The study proposed that a clear reimbursement policy should be presented to healthcare professionals, including pharmacists and patients, to resolve pharmacists' concerns regarding financial coverage. Also, measures to enhance the security of genomic results, such as using security technology, should be in place, and pharmacists need to be aware of these security measures to ensure data confidentiality. Furthermore, the study highlighted that laws such as the Genetic Information Nondiscrimination Act (USA) are in place to protect patients against discrimination by employers and medical/health insurance companies [34]. Overall, pharmacist's concerns should not be overlooked as they can hinder pharmacogenomics implementation.

The present study results showed that an increased number of patients and fewer prescriptions were associated with a more positive attitude toward pharmacogenomics testing. The positive impact of the increased number of patients could translate to better social and emotional interactions with the patients, especially in the case of elderly, immunocompromised people, or infants, hence the need for the testing. By extension, these community pharmacists are likely aware of their vital role in utilizing the testing results. For instance, if some medications were ineffective or resulted in side effects, having pharmacogenomics test results would resolve these situations or prevent them from occurring in the first place by the community pharmacist, hence the positive attitude. On the other hand, similar to the impact on the level of knowledge, increased prescriptions translate to a higher workload and hence may become a barrier to introducing tasks such as referring to the pharmacogenomic test results or producing ineffective relationships with doctors or nurses and negative attitudes. Hence, the increased workload due to a high number of prescriptions needs to be considered due to its association with poor attitudes and hence can pose an obstacle in integrating the testing service within the community pharmacy.

## Limitations

The study has some limitations. The cross-sectional design could not establish the cause-effect relationship. We have not assessed other factors, such as barriers to pharmacogenomics implementation or what support is needed to boost the self-confidence of pharmacists in order to recommend the test and interpret the test results to gain better insight. The convenience sampling technique might increase the risk of selection bias. However, the large sample size could minimize selection bias's effect on the study findings' accuracy and generalizability.

## Conclusion

The findings from this study suggest a veritable gap in knowledge regarding testing among community pharmacists in the UAE. There is a need for concerted efforts toward educational and training activities, particularly regarding pharmacogenomics testing for medications available in the UAE, the influence of genetic makeup on response to medications, and the indispensable use of pharmacogenomics in identifying potential drug–drug interactions. Furthermore, workload reduction can improve knowledge acquisition and, hence, the resolution of poor knowledge association with increased patient and prescription numbers.

Regarding attitudes, educational interventions must focus on guidance for testing in community pharmacies in an approach different from tertiary care settings. Also, workload reduction could significantly turn around negative attitudes toward the testing. Further transversal research involving other stakeholders, including physicians and genetic counselors, can suggest the best way forward.

## Data Availability

The datasets used and/or analyzed during the current study are available from the corresponding author on reasonable request.
